# Access to COVID-19 testing by individuals with housing insecurity during the early days of the COVID-19 pandemic in the United States: a scoping review

**DOI:** 10.3389/fpubh.2023.1237066

**Published:** 2023-09-28

**Authors:** Jon M. Johannesson, William A. Glover, Cathy A. Petti, Timothy H. Veldman, Ephraim L. Tsalik, Donald H. Taylor, Stephanie Hendren, Coralei E. Neighbors, L. Gayani Tillekeratne, Scott W. Kennedy, Barrie Harper, Warren A. Kibbe, Giselle Corbie, Michael Cohen-Wolkowiez, Christopher W. Woods, Mark J. Lee

**Affiliations:** ^1^Department of Medicine, Duke University School of Medicine, Durham, NC, United States; ^2^Duke Clinical Research Institute, Duke University School of Medicine, Durham, NC, United States; ^3^North Carolina State Laboratory of Public Health, North Carolina Department of Health and Human Services, Raleigh, NC, United States; ^4^Healthspring Global Inc., Bradenton, FL, United States; ^5^Duke Global Health Institute, Durham, NC, United States; ^6^Hubert-Yeargan Center for Global Health, Duke University, Durham, NC, United States; ^7^Sanford School of Public Policy, Duke University, Durham, NC, United States; ^8^Duke University Medical Center Library, Duke University, Durham, NC, United States; ^9^Department of Biostatistics and Bioinformatics, Duke University School of Medicine, Durham, NC, United States; ^10^Duke Cancer Institute, Duke University School of Medicine, Durham, NC, United States; ^11^Center for Health Equity Research, University of North Carolina, Chapel Hill, NC, United States; ^12^Department of Social Medicine, University of North Carolina, Chapel Hill, NC, United States; ^13^Department of Medicine, University of North Carolina, Chapel Hill, NC, United States; ^14^Department of Internal Medicine, University of North Carolina, Chapel Hill, NC, United States; ^15^Department of Pediatrics, Duke University School of Medicine, Durham, NC, United States; ^16^Department of Pathology, Duke University School of Medicine, Durham, NC, United States

**Keywords:** pandemic, COVID-19, healthcare disparities, inequities, healthcare barriers, underserved

## Abstract

**Introduction:**

The COVID-19 pandemic focused attention on healthcare disparities and inequities faced by individuals within marginalized and structurally disadvantaged groups in the United States. These individuals bore the heaviest burden across this pandemic as they faced increased risk of infection and difficulty in accessing testing and medical care. Individuals experiencing housing insecurity are a particularly vulnerable population given the additional barriers they face. In this scoping review, we identify some of the barriers this high-risk group experienced during the early days of the pandemic and assess novel solutions to overcome these barriers.

**Methods:**

A scoping review was performed following PRISMA-Sc guidelines looking for studies focusing on COVID-19 testing among individuals experiencing housing insecurity. Barriers as well as solutions to barriers were identified as applicable and summarized using qualitative methods, highlighting particular ways that proved effective in facilitating access to testing access and delivery.

**Results:**

Ultimately, 42 studies were included in the scoping review, with 143 barriers grouped into four categories: lack of cultural understanding, systemic racism, and stigma; medical care cost, insurance, and logistics; immigration policies, language, and fear of deportation; and other. Out of these 42 studies, 30 of these studies also suggested solutions to address them.

**Conclusion:**

A paucity of studies have analyzed COVID-19 testing barriers among those experiencing housing insecurity, and this is even more pronounced in terms of solutions to address those barriers. Expanding resources and supporting investigators within this space is necessary to ensure equitable healthcare delivery.

## 1. Introduction

Coronavirus disease 2019 (COVID-19) presented an unprecedented challenge across the United States and worldwide. Although the pandemic was a shared experience in the United States, COVID-19 has shined a spotlight on preexisting problems of healthcare inequity and disparities faced by individuals in structurally disadvantaged groups (1). COVID-19 disproportionately infected and affected individuals in marginalized communities, as indicated by the higher mortality and morbidity suffered by Black, Hispanic, and Native Americans compared with non-Hispanic White individuals, as well as by the economic struggles and food insecurities experienced by individuals living in poverty (1–3).

Individuals experiencing housing insecurity, many of whom already face structural disadvantages due to race/ethnicity or socioeconomic status, are at increased risk of exposure to COVID-19 and have less access to medical care (1, 4). Broadly, these are individuals experiencing homelessness, migrant workers for temporary or seasonal employment, individuals incarcerated in prison or jail, and immigrants temporarily detained at facilities. Individuals experiencing housing insecurity during a pandemic are faced with greater challenges in accessing information, getting tested, and receiving appropriate medical care. An estimated 6 million people in the United States experience housing insecurity annually. This sizable group consists of over 2 million incarcerated individuals (5), 30,000 refugees and asylum seekers (6), 3 million migrant and seasonal workers (7, 8), and half a million homeless individuals (9). Collectively, they add up to 6 million individuals, or almost 2% of the US population, and would rank in the top 20 most populous state based on the 2020–2022 US Census state ranking report (10).

In this article, we review publications regarding COVID-19 testing barriers experienced by these underserved groups and discuss efforts put forth by investigators to overcome some of these barriers. In our analysis, we included studies on the broader Latino migrant and immigrant community beyond those specifically focused on migrants in temporary housing and immigrants in detention facilities given the shared experiences within the broader community, and difficulties in teasing out subpopulations within these studies. Understanding these barriers will help not only to prepare for future pandemics but also to frame and address long-standing healthcare inequities and disparities in the United States.

## 2. Methods

### 2.1. Literature search methodology

To identify barriers in accessing COVID-19 testing experienced by individuals with housing insecurity, in collaboration with a medical librarian, we conducted an extensive publication search covering December 1, 2019, through April 4, 2022, on MEDLINE (PubMed), Embase (Elsevier), and CINAHL Complete (EBSCOhost) using a combination of keywords and database-specific subject headings for the following concepts: transient populations (migrants/immigrants, incarcerated, people experiencing housing instability), and non-traditional COVID-19 testing (defined as testing not conducted in a traditional healthcare setting) (Supplementary Table S1). No restrictions were placed on language. The search strategies were peer-reviewed by a second librarian with expertise in systematic review prior to the data/publication gathering. All citations from the search were uploaded to an EndNote 20 library before being uploaded to Covidence,^1^ which was used to handle the citations during primary and secondary review. Additional references were identified, as applicable, by hand-searching bibliographies of included articles.

### 2.2. Protocol and registration

A scoping review protocol was made and shared openly on the Open Science Framework,^2^ including the full search strategies.

### 2.3. Eligibility criteria

For primary review, all studies regardless of design or publication status, with full text in English directly or indirectly related to COVID-19 testing in individuals experiencing housing insecurity in the United States, were included. Populations considered for inclusion were (1) residents in prisons, jails, and detention centers; (2) people experiencing homelessness, including those in unstable housing (shelters and halfway homes); and (3) migrant/immigrant populations. To ensure inclusion of migrant/immigrant populations, studies were also included if they focused on rural populations, ethnic minorities, or those with potential language barriers. Search terms were also included that encompassed non-traditional COVID-19 diagnostic approaches; this included assays designed for supervised or unsupervised self-test or self-collection in any setting (e.g., home or clinic), or collection or test performed by a healthcare worker outside of a health system setting (e.g., community centers, mobile clinics, minute clinics). This was however not a factor in the inclusion or exclusion criteria. Study titles and abstracts were screened for eligibility in a primary review. If included, a study went to secondary review. For secondary review, studies were read in their entirety. To be included, the study had to include or summarize explicit quantitative or qualitative data about COVID-19 testing in populations of interest as defined above, which also included testing barriers. Review articles were not included in secondary review. Reasons for excluding trials were recorded.

### 2.4. Review and data collection

For the primary review, two independent reviewers screened titles and abstracts of all studies yielded from the search after exclusion of duplicates. Studies were included for secondary review if the aforementioned inclusion criteria were fulfilled. The full text of all studies was obtained and further analyzed by the same two reviewers in the secondary review. In all cases, if there was a disagreement regarding the inclusion or exclusion of a study, this was resolved via discussion between the two reviewers.

Data were extracted using standardized forms within Covidence and further confirmed by a separate reviewer. Extracted data included study name, study authors, date of publication, publication status, study type (with further subclassification), population studied, time period of study, study location and setting, exact details of the study, primary outcome and secondary outcome information, study participants, age of study participants, and all relevant clinical outcomes. Data collection was stratified. Data were stored in Covidence and exported into Excel.

The eligibility criteria were not limited by study type except for review articles, which were excluded. The eligibility criteria included studies from December 1, 2019, through April 4, 2022. Studies were limited to the United States to maximize comparability and increase generalizability of results for policy making. Study setting was documented as it applied to each study, including home, health system (outpatient clinic or hospital), or community. Studies were considered for inclusion and analysis regardless of publication status.

### 2.5. Identification and categorization of barriers to COVID-19 testing

Publications that mentioned or described barriers to COVID-19 testing experienced by any individual within the population of interest were further analyzed. Specific barriers were identified, grouped, and tabulated with some publications having multiple barriers.

## 3. Results

**Figure 1:**
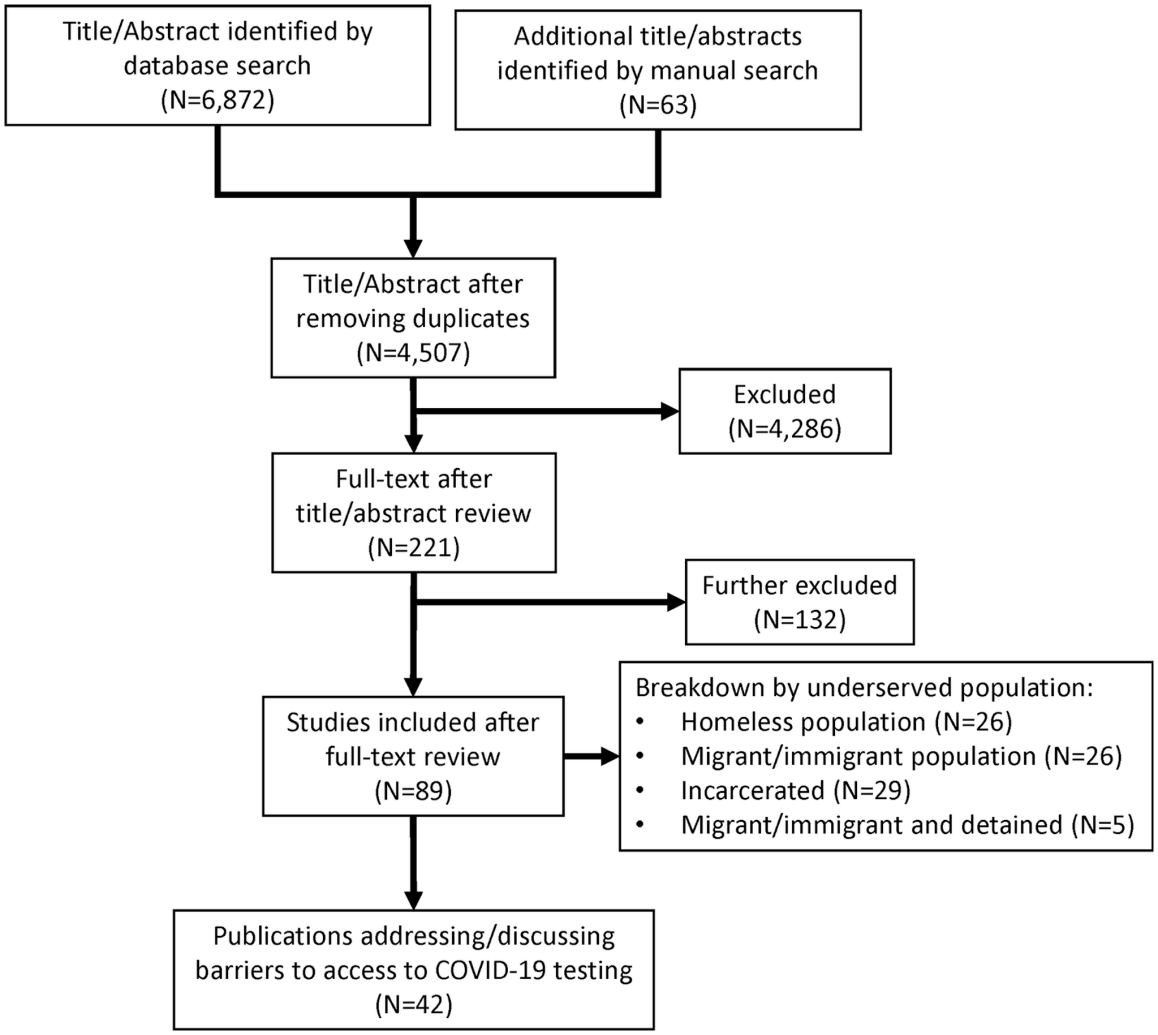
PRISMA flowchart. Literature search by database query (*N* = 6,872) and manual search (*N* = 63) covering December 1, 2019, through April 4, 2022, resulted in 42 publications of interest.

The initial query identified 6,872 publications, with 63 additional publications identified manually with review of reference lists. After removal of duplicate publications, title and abstract reviews, and full-text reviews, only 42 publications reported or discussed barriers to COVID-19 testing in our target population (11–53) (Figure 1). These studies have a wide range of populations, geographic locations, and settings reflecting the breadth of individuals facing housing insecurity and the complexity they face (Supplementary Table S2). Barriers were identified from data gathered through surveys, analysis of interviews, synthesis of prior publications, and commentaries from key opinion leaders. Most publications reported or described multiple barriers. We identified a total of 143 specific barriers grouped into four categories: Cultural Barriers; Immigration and Language Barriers; Insurance, Cost, and Logistic Barriers; and Other. These were broken down into respective underserved populations: Homeless; Immigrant/Migrant; Incarcerated; and Detained (i.e., immigration) (Table 1).

**Table 1:** List of barriers by groups.

Category	Barriers	Homeless	Immigrant/Migrant	Incarcerated	Detained
Cultural barriers	Lack of culturally sensitive approaches	1	5		
	Lack of trust	1	7		1
	No access or perceived lack of access		2	4	1
	Poor health literacy	1	3		
	Stigma and racism in healthcare	1	2	1	
Immigration and language barriers	Undocumented status		2		
	Fears of deportation or other immigration ramifications		11		
	Fear of public charge rule		4		
	General anti-immigration policies		1		
	Lack of identification		2		
	Limited English proficiency		13		1
Insurance, cost, and logistic barriers	Closure of clinics		1		
	Distance to testing site	1	4		
	Fear of losing work	1	7		
	Fear of food insecurity	1	2		
	Fear of dying in hospital		1		
	Fear of quarantine or isolation	1	2	4	
	Fear of contact tracing	1			
	Fear of contracting COVID-19	1	1		
	Fear of losing personal privileges	3		1	
	Fear of law enforcement	1	1		
	Worries about safety			1	
	Institutional barriers to accessing healthcare				1
	Lack of appointment information	1	2		
	Lack of connection to healthcare	1	2		
	Lack of insurance		8		
	Medical care costs		5	2	
	Lack of information	1	4		1
	Lack of technology	3			
	Lag in or lack of result reporting		2		
	No appointments or need for appointment		3		
Other	Constant movement within facility			1	
	Drug abuse		1		
	Lack of time	1	2		
	Need to sign a form				1
	Not severe enough symptoms or limited to symptoms		2		

### 3.1. Barriers to COVID-19 testing

#### 3.1.1. Barrier: lack of cultural understanding, systemic racism, and stigma

Among the four categories, 17 publications identified cultural understanding, racism, stigma, and health literacy as significant barriers. While these are vastly different, many studies combined multiple constituent elements, collectively shedding light on these barriers. Even before the COVID-19 pandemic, marginalized groups were facing stigma, racism, mistrust, and lack of cultural understanding as significant barriers to accessing healthcare (11, 18, 21). The pandemic not only exacerbated these barriers but also brought to the forefront the pervasiveness of health inequity due to these barriers. One area of particular focus for improvement is effective messaging.

Culturally and racially sensitive messaging can become effective vehicles in delivering healthcare. Messengers can significantly contribute to effective messaging in these communities. In a commentary by Kanamori et al., the authors synthesized data collected in their previous studies from experts comprising migrant farmworkers, Latino community leaders, and mental health professionals (12). The authors point to the value of *personalismo*, which refers to the preference of friendship with individuals of similar sociodemographic background, and *collectivism*, which refers to a cultural orientation that values close, nurturing, and supportive interpersonal relationships over individualistic behaviors and attitudes, as important sources of information and trust that are largely left out of the general public health messaging strategy. Instead, misinformation and mistrust can permeate through these channels, more pronounced when combined with fear and lack of access to information. Authors state that COVID-19 is heavily stigmatized within the community, and the misinformation cycle perpetuates the stigma. While this study focused on farmworkers, other studies on homeless and incarcerated individuals point to similar barriers in information and communication within racial context (43, 49). This highlights the importance of controlling the delivery and flow of information.

However, even in a prison environment where the flow of information can be controlled, tailoring messages around cultural and linguistic needs is crucial for successful participation, according to a study that looked at mass COVID-19 testing in 16 prisons (13). Although the authors did not explicitly state the reason for this need, they found that in at least two prisons, over 15% of inmates refused testing; they attributed some portion of it to the lack of cultural and linguistic considerations in messaging. Overall, these studies suggest factors around racial and cultural stigma and misunderstanding can be significant barriers to accessing healthcare and that mitigation requires a multipronged approach.

#### 3.1.2. Barrier: medical care cost, insurance, and logistics

Our analysis revealed a significant number of barriers associated with medical costs and logistics of getting tested. Twenty-five publications identified one or more barriers within this category (Table 1). Individuals experiencing housing insecurity are especially vulnerable to these types of challenges. Having adequate healthcare coverage or access to a primary care provider is not common to individuals in this target population. Citing prior studies showing low primary care visitation rates in individuals experiencing homelessness, Knight et al. highlighted that barriers to healthcare contribute to decreased acceptance of COVID-19 testing in this group (11). The researchers also pointed out that limited access to the internet or telephones can lead to decreased knowledge about testing options, which can contribute to lower rates of testing. Likewise, in a perspective article, Behbahani et al. pointed out that while strategies like drive-thru testing have been largely successful in the general population, immigrants and people experiencing homelessness do not readily have access to motor vehicles to get to these sites (14).

Similarly, seasonal and migrant workers contracted to a particular farm or facility have limited access to primary care, and for most, health insurance is not provided by employers. Lauzardo et al. found that among the 100 migrant farmworkers in Florida involved in an outbreak, most reported having some COVID-19-related symptoms but could not seek medical care (53). Upon testing by the local public health department, 91 of the 100 workers tested positive for SARS-CoV-2. Based on the local health department investigation, the outbreak started with two workers who tested positive a week prior after presenting with symptoms.

Migrants and immigrants in the general community may also experience a similar lack of access to healthcare. During a free COVID-19 testing event in a predominantly Latino community in San Francisco, a survey was conducted to understand why participants did not seek testing prior to the free event (15). Some of the responses included not being able to get an appointment (25%), not knowing where or how to make an appointment (14%), not having insurance or a doctor (14%), and testing sites being too far away (3%). Many of these barriers are related to and affected by other barriers, such as linguistic challenges, access to transportation, and health literacy.

Collectively, these studies show how medical care cost, insurance, and logistics to access medical care present as barriers for individuals experiencing housing insecurity. Importantly, these studies also show that barriers do not exist in isolation but can be intertwined and codependent. Identifying and evaluating barriers, especially in these vulnerable populations, may be complex and necessitate complicated analysis in decoupling and understanding the association among and between barriers.

#### 3.1.3. Barrier: immigration policies, language, and fear of deportation

Migrants and immigrants face an additional complexity in accessing COVID-19 testing. Fifteen publications addressed barriers experienced by migrants and immigrants, including undocumented status. Being undocumented, whether through lapse of legal status (e.g., expired work permit) or undocumented entry to the United States, is a major barrier to medical care. Even for those with legal status, the prospect of deportation or dealing with harsh immigration policies, like the *public charge rule*, present as significant barriers (17). According to the public charge rule, a noncitizen who primarily depends on government assistance for subsistence, and thereby becomes a public charge, can be denied permanent residency (17). These challenges, combined with language and cultural barriers, place migrants and immigrants in a uniquely vulnerable position.

In one study, investigators found that the public charge rule is not only a significant concern but a major barrier to seeking medical care (18). Lechuga et al. conducted interviews through online surveys or by telephone, including with migrants and farmworkers. Among the participants, 76% reported not having health insurance, and 50.3% reported not seeking medical care in the United States in the preceding 12 months. Of the 49.3% who received medical care in the United States, 25.6% sought care at a hospital emergency room. When asked whether they would seek medical care if the clinic or doctor assured them that COVID-19-related care would not impact their immigration status or chances, 53.5% of participants were skeptical and would decline.

Cultural and linguistic barriers are additional challenges faced by migrant and immigrant populations. Davlantes et al. conducted a survey as well as interviews with key informants and several focus group sessions in Latino communities in the Prince William Health District in northern Virginia (20). When asked where they get their COVID-19 information, participants mentioned television (Spanish-language) and the Internet as primary sources, with clinics and hospitals being secondary. However, many participants indicated that health ministries from their countries of origin and prominent Hispanic or Latino business owners were also sources of information, underscoring the influence that “alternative” sources can have in this group. Participants consistently emphasized that most COVID-19 information was in English and was text-heavy, with less emphasis on videos, images, and graphics. Taken together, these studies underscore the unique situation that migrants and immigrants face in accessing medical care and emphasize how the pandemic should shift our focus on these barriers.

### 3.2. Potential solutions to overcome COVID-19 testing barriers

In addition to identifying barriers, 30 of the 42 publications we analyzed offered some potential solutions, and in a few studies, investigators implemented solutions aimed at overcoming certain barriers (16, 21, 22). Barriers to accessing medical care rooted in racism, mistrust, and lack of cultural understanding are systemic and difficult to overcome. Similarly, barriers based on fear of deportation or loss of privileges, financial burdens including lack of insurance or loss of employment, or logistical challenges such as transportation or telecommunication can be equally difficult to overcome. Many of these barriers are related to or dependent upon each other, making assessment and strategizing difficult. However, the strong public interest and motivation to curb the COVID-19 pandemic has presented us with unique opportunities to design and implement some innovative solutions. In our analysis, we identified three notable approaches, each uniquely targeted to a specific underserved population group (16, 21, 22). While we do not endorse a particular approach, these studies show that innovative approaches to overcome barriers can positively affect members of these underserved groups and the community at large.

#### 3.2.1. Delivering integrated care where it is needed: the backpack medicine program approach

During the course of the pandemic, widespread use of drive-thru testing sites, mobile testing units, and walk-in clinics at local pharmacies and national chain stores addressed gaps in meeting the demand for COVID-19 testing. For the most part, these outside-the-box solutions to alleviate test volumes in clinics and hospitals met the needs of the general public. However, people experiencing homelessness still face barriers that limit their access to these solutions, such as not having a motor vehicle to get to drive-thru testing sites, proper identification when required, or a way of receiving test results, as well as not knowing these testing options exist (11, 14, 15, 19, 20). Thus, people experiencing homelessness may require further creative approaches.

In one study, a team of medical professionals at the Ventura County Medical Center in Ventura, CA, expanded on an existing outreach program called Backpack Medicine Program (BMP) to include COVID-19 testing (21). The program began in 2018 to provide free outreach by bringing medical care to individuals experiencing homelessness where they are located, including homeless encampments and shelters, parks, and under freeways. In some instances, the program also delivered care to local farm workers. The program provided basic primary care, wound care, behavioral health and addiction medicine, and housing and benefit assistance, taking an integrated care approach. In response to the pandemic, anyone suspected of COVID-19 was offered testing. While waiting for test results or if results were positive, individuals were offered to isolate at a sponsored hotel free of charge until eligible to stop isolation. If they declined relocation, they were asked to quarantine away from others in their respective encampments. In the first 4 weeks of the program, over 150 individuals were tested, identifying 24 positive cases.

Although this was a small study, it shows one approach to bridging the gaps in health inequalities and providing integrated care to individuals who might not otherwise receive proper care. This approach addresses many of the barriers that limit access to medical care for people experiencing homelessness. However, there are some limitations to this approach. First, while the authors do not mention the cost associated with running this program, funding for implementing and maintaining this type of program on a larger, national scale may be cost-prohibitive. Second, the authors mentioned that police presence is needed on occasion due to safety concerns of the BMP providers. Although the local police department provided support in this particular study, it cannot be assumed that all police departments would be able to provide support. Lastly, given the sensitivity around the presence of homeless people, potential backlash within the larger community cannot be ignored. Despite these caveats, this approach has been shown to work effectively in this population group. A targeted, well-supported rollout of similar programs in other jurisdictions could start to build confidence in their effectiveness and lead to wider implementation. Over time, delivery of integrated medical care to vulnerable populations could become a more routine practice.

#### 3.2.2. Identifying cases in congregate settings before an outbreak: cohort-based testing approach

Individuals in congregate living situations, such as those incarcerated or detained in a facility, face similar challenges as those who experience homelessness, but with added complexity due to their proximity and confined living situation, in which a single positive case can quickly lead to an outbreak. Promptly identifying cases and implementing isolation and quarantine measures while providing necessary support and assurance to the affected individuals is crucial to preventing outbreaks. Symptom-based identification of cases is one common approach, but given certain barriers around reporting symptoms, including fear of losing privileges and financial consequences, it may not be the most effective strategy. Also, presymptomatic or asymptomatic transmission of SARS-CoV-2 would not be identified in this approach. The constant emergence of variants and our changing understanding of transmission of this virus adds an additional layer of complexity.

To address these challenges, a group of investigators used a cohort-based testing approach to promptly identify presymptomatic and asymptomatic cases in a correctional facility in Chicago (16). Wadhwa et al. conducted COVID-19 testing and interviews of exposed contacts of laboratory-confirmed cases (16). Individuals with a laboratory-confirmed COVID-19 infection were moved from their unit into an isolation unit, while the remaining exposed contacts were quarantined together in their respective units. The investigators approached 224 exposed detainees assigned to two groups: serial testing vs. single test on day 14. Those in the serial testing group were tested on days 1, 3–5, and 14 from the time of the first laboratory-confirmed case. Those in the 14-day group were only tested on day 14 to leave quarantine. In the serial testing group, 16 out of 96 persons tested positive on day 1, and one person tested positive on day 3–5, for a total of 17 positive cases. No one in the serial group tested positive on day 14. In the single test group, 2 out of 82 persons tested positive on day 14. Of the 19 positive cases, 12 (63%) were asymptomatic, and 4 (21%) were presymptomatic. These findings suggest that symptom-based testing alone would have missed 84% of the cases and could have led to greater transmission. Thus, cohort-based testing by serial testing, especially soon after identification of a positive case, could help reduce transmission in congregate settings.

Cohort-based testing in correctional facilities poses various limitations in implementation. Wadhwa et al. observed high rates of refusal to participate in the study due to fear of losing privileges in the form of the commissary or phone calls. After all, if not for the testing, the 12 individuals with asymptomatic cases would not have been confined in isolation and presumably have enjoyed privileges sooner. Therefore, while cohort-based testing is an effective approach to identifying cases and potentially preventing or reducing outbreaks (particularly in settings such as nursing homes), other barriers in the incarcerated/detainee setting must be addressed to ensure noncoercive participation. This could include providing access to phone calls in isolation or delivery of commissary items to isolation cells or units.

#### 3.2.3. Utilizing cultural and community centers as one-stop shops: community-adapted approach

Individuals within migrant and immigrant communities can face a wide range of challenges when attempting to access COVID-19 testing, from lack of access to a vehicle to fear of deportation. In this underserved population, an individual who is undocumented is liable to fear that contact tracing or any government involvement could lead to deportation. Engaging individuals within this group who are not only resource-limited but fear for their safety and liberty is a monumental task. However, as evidenced by the COVID-19 infection and mortality rates in this population, reaching members of underserved communities is critical.

One study took a community-adapted approach in addressing this need (22). The investigators partnered with the local health department and a community cultural center to develop a safe, culturally tailored space for COVID-19 testing designed to accommodate the specific needs of the local Latino community and other underserved populations, including sexual and gender minorities. The center is well known to the locals and is a gathering space for artist exhibitions, performances, religious services, and community gatherings. The program offers walk-in, drive-thru, and walk-up testing at no cost for those without insurance, regardless of symptom or in-state residency status. Additionally, there are onsite language services in English, Spanish, and Portuguese with additional languages via tele-interpretation.

To engage the community, the investigators worked with Latino community leaders and others to promote the program via social media, radio, churches, and other platforms. In the 2 months of the study, the program tested 498 participants, with 40% identifying as Latino, 32% as LGBTQIA+, and 52% as women. An important result of this study is that 90% of participants were asymptomatic. While the authors do not mention follow-up on these participants, one could assume that positive test results of these asymptomatic individuals may have reduced transmission in their respective communities or to their familial contacts. The investigators did not report on how the participants received the program. However, they mentioned that the program attracted individuals across the state, underscoring the success and need for this type of program.

Community-adapted testing programs using a well-known cultural center and engaging community leaders to provide testing services to marginalized groups that would otherwise remain disengaged can be an effective solution. This type of approach overcomes many barriers that other studies have identified. Although this framework can be useful, there are two caveats to this approach. First, implementation requires sustainable and consistent funding. Given that a testing site is required that needs to be staffed, along with substantial work in identifying and collaborating with local community leaders, the upfront investment may be substantial. In fact, the investigators noted that the human and financial resources needed to implement and maintain this type of testing site could limit the broader implementation of this type of program. Second, migrants and immigrants are not a monolithic group. Rather, they represent a spectrum of cultural backgrounds and experiences that cannot be fit into a single box of solutions. Therefore, this approach should be broadly defined, and the framework should be loosely structured, requiring adjustments and revisions at each specific implementation site. Nevertheless, this study shows that this approach can be successful and engage vulnerable populations.

## 4. Discussion and conclusion

The disproportionate impact of an infectious disease on morbidity and mortality in underserved populations is perhaps the ultimate proof of the impact of systemic or structural inequalities on human health. The COVID-19 pandemic reaffirmed how interconnected and interdependent we are with each other, at all levels of the socioeconomic ladder, across racial and ethnic backgrounds, and at varying geographic locations.

Our scoping review revealed that there are many barriers faced by individuals with housing insecurity, including those in congregate living situations, temporary or transient living situations, other non-traditional settings, and individuals within other marginalized groups facing barriers in accessing COVID-19 testing. These individuals were not only left behind in major efforts to curb the virus, but also carried the heaviest burden of the pandemic. While individuals in congregate living situations are at risk of acquiring COVID-19 from others within the facilities, individuals in temporary or transient living situations and those living on streets, bridges, and other outdoor settings are not only exposed to acquiring COVID-19 as they move around, but also face challenges in accessing information, medical care, protective devices, and testing information that may be available in congregate facilities.

While there are many barriers, our scoping review also revealed that there are potential solutions. Several studies have implemented some of these solutions in small scale, while others have provided proposals and call-to-action solutions derived from various bodies of evidence. In our assessment, many of these solutions, especially those that include direct outreach and engagement with individuals in various levels and stages of housing insecurity, require tremendous investment. Creating community centers, empowering local/community leaders who can build trust and disseminate appropriate messaging, providing “house” calls for medical care, and building an overall infrastructure where everyone has equal access to testing require close coordination with insurance companies, healthcare organizations, government agencies, and strong political will.

Identifying barriers, determining root causes, and developing approaches to overcome these barriers are paramount in bridging healthcare inequity and disparity, for both future pandemics and overall delivery of medical care. Given the paucity of publications focusing on barriers faced by marginalized groups, there is a great need to expand resources and support investigators within this sphere. One national effort has recently come from the National Institutes of Health through the Rapid Acceleration of Diagnostics–Underserved Populations (RADx-UP) grant program, where studies (including this scoping review) are funded to expand COVID-19 testing and gather data that could inform potential barriers and solutions in marginalized communities.

Studies focused on distilling each of these barriers with study designs to understand the needs of those with housing insecurity are critically needed, especially studies that further focus on subpopulations including age (i.e., youth and older adult), gender, and wider ethnic groups. Studies with sufficient funding to carry out larger-scale and long-term implementation of solutions like delivery of medical care where the need is, or building community centers and infrastructure, are also needed. These studies are crucial in further assessing the issue and experimenting with different solutions. They are also needed to get wider commitment and buy-in from respective governmental entities (federal and local), healthcare facilities and payors, and community groups, and ultimately needed to provide realistic and sustaining solutions.

## Data availability statement

The raw data supporting the conclusions of this article will be made available by the authors, without undue reservation.

## Author contributions

JJ, WG, CP, TV, ET, CN, LT, SK, BH, CW, and ML: conceptualization. JJ, CP, TV, ET, CW, and ML: methodology and analysis. JJ and ML: original draft. JJ, SH, and ML: data search and extraction. JJ, WG, CP, TV, ET, DT, CN, LT, SK, BH, WK, GC, MC-W, CW, and ML: review and editing. MC-W and CW: supervision. All authors contributed to the article and approved the submitted version.

## Funding

Research reported in this Rapid Acceleration of Diagnostics–Underserved Populations (RADx-UP) publication was supported by the National Institutes of Health (Award No. U24MD016258). The content was solely the responsibility of the authors and does not necessarily represent the official views of the National Institutes of Health.

## Conflict of interest

CP was employed by the Healthspring Global Inc. CP reports receiving consulting fees from Abbott Molecular and Rapid Diagnostics. ET has been a consultant for Biomeme, Inc., has a patent pending for Methods to Diagnose and Treat Acute Respiratory Infections (US20180245154A1), and is currently employed by Danaher Corp. MC-W reports receiving support for research from the NIH (1U24-MD016258), National Institute of Allergy and Infectious Diseases (HHSN272201500006I, 1K24-AI143971), U.S. Food and Drug Administration (5U18-FD006298), and industry for drug development in adults and children. CW reports grants or contracts from DARPA, NIH-ARLG/NIAID/VTEU/NIMHO/NIGMS, Sanofi, Najit, CDC, Patient-Centered Outcomes Research Institute, USAMRAA, DOD, Abbott, and Pfizer; consulting fees from Arena Pharmaceuticals, BioFire, FHI Clinical, Giner, Karius, and SeLux Diagnostics; support for attending meetings and/or travel from American Society for Microbiology; patents planned, issued or pending for: biomarkers for the molecular classification of bacterial infection, methods to diagnose and treat acute respiratory infections, gene expression signatures useful to predict or diagnose sepsis and methods of using the same, host based molecular signatures of human infection with SARS-CoV-2 (COVID19), methods of identifying infectious disease and assays for identifying infectious disease, and nasopharyngeal protein biomarkers of acute respiratory virus infection and methods of using same; participation on a Data Safety Monitoring Board or Advisory Board for IDbyDNA, Janssen, Regeneron, Roche Molecular Sciences; leadership or fiduciary role in other board, society, committee or advocacy group, paid or unpaid for American Society for Microbiology and American Society of Tropical Medicine and Hygiene; and other financial or non-financial interests with Biomeme and Predigen, Inc.

The remaining authors declare that the research was conducted in the absence of any commercial or financial relationships that could be construed as a potential conflict of interest.

## Publisher’s note

All claims expressed in this article are solely those of the authors and do not necessarily represent those of their affiliated organizations, or those of the publisher, the editors and the reviewers. Any product that may be evaluated in this article, or claim that may be made by its manufacturer, is not guaranteed or endorsed by the publisher.

## Footnotes

^1^ https://app.covidence.org/

^2^ https://osf.io/xe27b/
