# Supplementary material for: Access to COVID-19 testing by individuals with housing insecurity during the early days of the COVID-19 pandemic in the United States: a scoping review
Source: Front Public Health. 2023 Sep 28;11:1237066. doi: 10.3389/fpubh.2023.1237066 (PMC10568314; doi:10.3389/fpubh.2023.1237066)
Supplement: Supplementary file 1 [file Table_1.DOCX]

**Table S1A. Database query search keyword: MEDLINE (PubMed)**

| **Set #** | **Search Strategy** | **Results** |
| --- | --- | --- |
| 1  *Population terms* | "Vulnerable Populations"[Mesh] OR "Public Housing"[Mesh] OR "Homeless Persons"[Mesh] OR "Homeless Youth"[Mesh] OR "Refugee Camps"[Mesh] OR "Refugees"[Mesh] OR "Emigration and Immigration"[Mesh] OR "Transients and Migrants"[Mesh] OR "Human Migration"[Mesh] OR "Undocumented Immigrants"[Mesh] OR Correctional Facilities[Mesh] OR "Prisons"[Mesh] OR Jails[Mesh] OR "Prisoners"[Mesh] OR Homeless[tiab] OR homelessness[tiab] OR "unstable housing"[tiab] OR "housing instability"[tiab] OR "street people"[tiab] OR Refugee[tiab] OR refugees[tiab] OR immigrant[tiab] OR immigrants[tiab] OR immigration[tiab] OR immigrate[tiab] OR immigrated[tiab] OR immigrates[tiab] OR immigrating[tiab] OR emigration[tiab] OR emigrants[tiab] OR emigrant[tiab] OR emigrating[tiab] OR emigrated[tiab] OR emigrates[tiab] OR emigrate[tiab] OR migrants[tiab] OR migrant[tiab] OR migration[tiab] OR migrated[tiab] OR migrates[tiab] OR migrate[tiab] OR "border crossing"[tiab] OR "border crossings"[tiab] OR settlement[tiab] OR settlements[tiab] OR resettlement[tiab] OR resettlements[tiab] OR undocumented[tiab] OR alien[tiab] OR aliens[tiab] OR foreign[tiab] OR foreigners[tiab] OR foreigner[tiab] OR "displaced person"[tiab] OR "displaced persons"[tiab] OR transient[tiab] OR transients[tiab] OR "seasonal worker"[tiab] OR "seasonal workers"[tiab] OR “correctional facility”[tiab] OR “correctional facilities”[tiab] OR “penal institution”[tiab] OR “penal institutions”[tiab] OR “correctional institution”[tiab] OR “correctional institutions”[tiab] OR “detention center”[tiab] OR “detention centers”[tiab] OR Prison[tiab] OR prisons[tiab] OR penitentiary[tiab] OR penitentiaries[tiab] OR prisoner[tiab] OR prisoners[tiab] OR imprison[tiab] OR imprisoned[tiab] OR imprisons[tiab] OR imprisoning[tiab] OR imprisonment[tiab] OR jail[tiab] OR jails[tiab] OR jailed[tiab] OR jailing[tiab] OR jailer[tiab] OR jailers[tiab] OR inmate[tiab] OR inmates[tiab] OR incarceration[tiab] OR incarcerations[tiab] OR incarcerated[tiab] OR incarcerate[tiab] OR incarcerates[tiab] OR incarcerating[tiab] OR "vulnerable person"[tiab] OR "vulnerable persons"[tiab] OR "vulnerable population"[tiab] OR "vulnerable populations"[tiab] | 892540 |
| 2  *Covid testing* | "COVID-19 Serological Testing"[Mesh] OR "COVID-19 Nucleic Acid Testing"[MeSH] OR "COVID-19 Testing"[MeSH] | 8535 |
| 3  *Covid* | "COVID-19"[MeSH] OR "SARS-CoV-2"[MeSH] OR "COVID-19"[Supplementary Concept] OR "severe acute respiratory syndrome coronavirus 2"[Supplementary Concept] OR "COVID-19 breakthrough infections" [Supplementary Concept] OR "SARS-CoV-2 variants" [Supplementary Concept] OR COVID[tw] OR "COVID-19"[tw] OR "COVID 19"[tw] OR COVID19[tw] OR 2019nCov[tw] OR "2019-nCoV"[tw] OR "2019 ncov"[tw] OR SARS-CoV-2[tw] OR SARSCov2[tw] OR SARS-CoV2[tw] OR (((Wuhan[tw] Or Hubei[tw] OR Huanan[tw]) AND coronavirus[tw]) OR ((new[tw] OR novel[tw]) AND coronavirus[tw]) AND English[lang]) | 243402 |
| 4  *Testing* | "Self-Testing"[Mesh] OR "Direct-To-Consumer Screening and Testing"[Mesh] OR "Quick Diagnosis Units"[Mesh] OR test*[tiab] OR self-test*[tiab] OR "self-collect*"[tiab] OR "self-administrat*"[tiab] OR BinaxNOW[tiab] OR CRISPR[tiab] OR "non-clinical"[tiab] OR "non clinical"[tiab] OR sample[tiab] OR samples[tiab] OR administrat*[tiab] OR "mail-in"[tiab] OR "mail in"[tiab] OR "mailed in"[tiab] OR drive-thru[tiab] OR "drive thru"[tiab] OR drive-thrus[tiab] OR "drive thrus"[tiab] OR "drive through"[tiab] OR drive-through[tiab] OR drive-throughs[tiab] OR "drive throughs"[tiab] OR drive-in[tiab] OR "drive in"[tiab] OR "quick diagnosis unit"[tiab] OR "quick diagnosis units"[tiab] OR walk-in[tiab] OR "walk in"[tiab] OR walk-ins[tiab] OR "walk ins"[tiab] OR test*[tiab] OR diagnos*[tiab] OR screen*[tiab] OR sampl*[tiab] OR collect*[tiab] OR administrat*[tiab] | 9261743 |
| 5 | 2 OR (3 AND 4) | 93512 |
| 6 | 1 AND 5 | 2606 |
| 7 | 6 AND ("2019/12/01"[Date - MeSH] : "3000"[Date - MeSH]) | 2581 |

**Table S1B. Database query search keyword: Embase (Elsevier)**

| **Set #** | **Search Strategy** | **Results** |
| --- | --- | --- |
| 1  *Population terms* | 'vulnerable population'/exp OR 'disadvantaged population'/exp OR 'housing'/exp OR 'homeless person'/exp OR 'homeless man'/exp OR 'homeless woman'/exp OR 'homeless youth'/exp OR 'refugee camp'/exp OR 'refugee'/exp OR 'migration'/exp OR 'forced migration'/exp OR 'immigration'/exp OR 'migration'/exp OR 'undocumented immigrant'/exp OR 'correctional facility'/exp OR (Homeless OR homelessness OR 'unstable housing' OR 'housing instability' OR 'street people' OR Refugee OR refugees OR immigrant OR immigrants OR immigration OR immigrate OR immigrated OR immigrates OR immigrating OR emigration OR emigrants OR emigrant OR emigrating OR emigrated OR emigrates OR emigrate OR migrants OR migrant OR migration OR migrated OR migrates OR migrate OR 'border crossing' OR 'border crossings' OR settlement OR settlements OR resettlement OR resettlements OR undocumented OR alien OR aliens OR foreign OR foreigners OR foreigner OR 'displaced person' OR 'displaced persons' OR transient OR transients OR 'seasonal worker' OR 'seasonal workers' OR 'correctional facility' OR 'correctional facilities' OR 'penal institution' OR 'penal institutions' OR 'correctional institution' OR 'correctional institutions' OR 'detention center' OR 'detention centers' OR Prison OR prisons OR penitentiary OR penitentiaries OR prisoner OR prisoners OR imprison OR imprisoned OR imprisons OR imprisoning OR imprisonment OR jail OR jails OR jailed OR jailing OR jailer OR jailers OR inmate OR inmates OR incarceration OR incarcerations OR incarcerated OR incarcerate OR incarcerates OR incarcerating OR 'vulnerable person' OR 'vulnerable persons' OR 'vulnerable population' OR 'vulnerable populations'):ti,ab | 1144321 |
| 2  *Covid testing* | 'COVID-19 serological testing'/exp OR 'COVID-19 testing'/exp OR 'COVID-19 nucleic acid testing'/exp | 5679 |
| 3  *Covid* | 'coronavirus disease 2019'/exp OR 'Severe acute respiratory syndrome coronavirus 2'/exp OR (COVID OR 'COVID-19' OR 'COVID 19' OR COVID19 OR 2019nCov OR '2019-nCoV' OR '2019 ncov' OR SARS-CoV-2 OR SARSCov2 OR SARS-CoV2 OR ((Wuhan Or Hubei OR Huanan) AND coronavirus) OR ((new OR novel) AND coronavirus)):ti,ab,kw | 268309 |
| 4  *Testing* | 'viral load testing'/exp OR 'self-testing'/exp OR 'screening test'/exp OR 'quick diagnosis unit'/exp OR (test* OR self-test* OR 'self-collect*' OR 'self-administrat*' OR BinaxNOW OR CRISPR OR 'non-clinical' OR 'non clinical' OR sample OR samples OR administrat* OR 'mail-in' OR 'mail in' OR 'mailed in' OR drive-thru OR 'drive thru' OR drive-thrus OR 'drive thrus' OR 'drive through' OR drive-through OR drive-throughs OR 'drive throughs' OR drive-in OR 'drive in' OR drive-ins OR 'drive ins' OR walk-in OR 'walk in' OR walk-ins OR 'walk ins' OR diagnos* OR screen* OR sampl* OR collect* OR administrat*):ti,ab | 12767040 |
| 5 | 2 OR (3 AND 4) | 108201 |
| 6 | 1 AND 5 | 3544 |
| 7 | 6 AND [12-01-2019]/sd | 3524 |

**Table S1C. Database query search keyword: CINAHL Complete (EBSCOhost)**

| **Set #** | **Search Strategy** | **Results** |
| --- | --- | --- |
| 1  *Population terms* | (MH "Special Populations") OR (MH "Public Housing") OR (MH "Homeless Persons") OR (MH "Homelessness") OR (MH "Refugee Camps") OR (MH "Refugees+") OR (MH "Emigration and Immigration") OR (MH "Transients and Migrants") OR (MH "Undocumented Immigrants") OR (MH "Immigrants+") OR (MH "Emigration and Immigration") OR (MH "Correctional Facilities") OR (MH "Correctional Facilities") OR (MH "Prisoners") OR TI (Homeless OR homelessness OR "unstable housing" OR "housing instability" OR "street people" OR Refugee OR refugees OR immigrant OR immigrants OR immigration OR immigrate OR immigrated OR immigrates OR immigrating OR emigration OR emigrants OR emigrant OR emigrating OR emigrated OR emigrates OR emigrate OR migrants OR migrant OR migration OR migrated OR migrates OR migrate OR "border crossing" OR "border crossings" OR settlement OR settlements OR resettlement OR resettlements OR undocumented OR alien OR aliens OR foreign OR foreigners OR foreigner OR "displaced person" OR "displaced persons" OR transient OR transients OR "seasonal worker" OR "seasonal workers" OR “correctional facility” OR “correctional facilities” OR “penal institution” OR “penal institutions” OR “correctional institution” OR “correctional institutions” OR “detention center” OR “detention centers” OR Prison OR prisons OR penitentiary OR penitentiaries OR prisoner OR prisoners OR imprison OR imprisoned OR imprisons OR imprisoning OR imprisonment OR jail OR jails OR jailed OR jailing OR jailer OR jailers OR inmate OR inmates OR incarceration OR incarcerations OR incarcerated OR incarcerate OR incarcerates OR incarcerating OR "vulnerable person" OR "vulnerable persons" OR "vulnerable population" OR "vulnerable populations") OR AB (Homeless OR homelessness OR "unstable housing" OR "housing instability" OR "street people" OR Refugee OR refugees OR immigrant OR immigrants OR immigration OR immigrate OR immigrated OR immigrates OR immigrating OR emigration OR emigrants OR emigrant OR emigrating OR emigrated OR emigrates OR emigrate OR migrants OR migrant OR migration OR migrated OR migrates OR migrate OR "border crossing" OR "border crossings" OR settlement OR settlements OR resettlement OR resettlements OR undocumented OR alien OR aliens OR foreign OR foreigners OR foreigner OR "displaced person" OR "displaced persons" OR transient OR transients OR "seasonal worker" OR "seasonal workers" OR “correctional facility” OR “correctional facilities” OR “penal institution” OR “penal institutions” OR “correctional institution” OR “correctional institutions” OR “detention center” OR “detention centers” OR Prison OR prisons OR penitentiary OR penitentiaries OR prisoner OR prisoners OR imprison OR imprisoned OR imprisons OR imprisoning OR imprisonment OR jail OR jails OR jailed OR jailing OR jailer OR jailers OR inmate OR inmates OR incarceration OR incarcerations OR incarcerated OR incarcerate OR incarcerates OR incarcerating OR "vulnerable person" OR "vulnerable persons" OR "vulnerable population" OR "vulnerable populations") | 156479 |
| 2  *Covid testing* | (MH "COVID-19 Testing") OR (MH "SARS-CoV-2") | 2289 |
| 3  *Covid* | TI (COVID OR "COVID-19" OR "COVID 19" OR COVID19 OR 2019nCov OR "2019-nCoV" OR "2019 ncov" OR SARS-CoV-2 OR SARSCov2 OR SARS-CoV2 OR ((Wuhan Or Hubei OR Huanan) AND coronavirus) OR ((new OR novel) AND coronavirus)) OR AB (COVID OR "COVID-19" OR "COVID 19" OR COVID19 OR 2019nCov OR "2019-nCoV" OR "2019 ncov" OR SARS-CoV-2 OR SARSCov2 OR SARS-CoV2 OR ((Wuhan Or Hubei OR Huanan) AND coronavirus) OR ((new OR novel) AND coronavirus)) | 79845 |
| 4  Testing | (MH "Self-Testing") OR TI (test* OR self-test* OR "self-collect*" OR "self-administrat*" OR BinaxNOW OR CRISPR OR "non-clinical" OR "non clinical" OR sample OR samples OR administrat* OR "mail-in" OR "mail in" OR "mailed in" OR drive-thru OR "drive thru" OR drive-thrus OR "drive thrus" OR "drive through" OR drive-through OR drive-throughs OR "drive throughs" OR drive-in OR "drive in" OR "quick diagnosis unit" OR "quick diagnosis units" OR walk-in OR "walk in" OR walk-ins OR "walk ins" OR test* OR diagnos* OR screen* OR sampl* OR collect* OR administrat*) OR AB (test* OR self-test* OR "self-collect*" OR "self-administrat*" OR BinaxNOW OR CRISPR OR "non-clinical" OR "non clinical" OR sample OR samples OR administrat* OR "mail-in" OR "mail in" OR "mailed in" OR drive-thru OR "drive thru" OR drive-thrus OR "drive thrus" OR "drive through" OR drive-through OR drive-throughs OR "drive throughs" OR drive-in OR "drive in" OR "quick diagnosis unit" OR "quick diagnosis units" OR walk-in OR "walk in" OR walk-ins OR "walk ins" OR test* OR diagnos* OR screen* OR sampl* OR collect* OR administrat*) | 1875695 |
| 5 | 2 OR (3 AND 4) | 23185 |
| 6 | 1 AND 5 | 766 |
| 7 | Date filtered to: 2019 - present | 765 |
